# Overdosage of *HNF1B* Gene Associated With Annular Pancreas Detected in Neonate Patients With 17q12 Duplication

**DOI:** 10.3389/fgene.2021.615072

**Published:** 2021-05-07

**Authors:** Feifan Xiao, Xiuyun Liu, Yulan Lu, Bingbing Wu, Renchao Liu, Bo Liu, Kai Yan, Huiyao Chen, Guoqiang Cheng, Laishuan Wang, Qi Ni, Gang Li, Ping Zhang, Xiaomin Peng, Yun Cao, Chun Shen, Huijun Wang, Wenhao Zhou

**Affiliations:** ^1^Center for Molecular Medicine, Children’s Hospital of Fudan University, National Children’s Medical Center, Institutes of Biomedical Sciences, Fudan University, Shanghai, China; ^2^Center for Molecular Medicine, Children’s Hospital of Fudan University, National Children’s Medical Center, Shanghai, China; ^3^Division of Neonatology, Key Laboratory of Neonatal Diseases, Ministry of Health, Children’s Hospital of Fudan University, National Children’s Medical Center, Shanghai, China; ^4^Department of Pediatric Surgery, Children’s Hospital of Fudan University, National Children’s Medical Center, Shanghai, China

**Keywords:** annular pancreas, 17q12 duplication, CNV, *HNF1B*, zebrafish

## Abstract

The annular pancreas (AP) is a congenital anomaly of the pancreas that can cause acute abdominal pain and vomiting after birth. However, the genetic cause of AP is still unknown, and no study has reported AP in patients with 17q12 duplication. This study retrospectively analyzed the next-generation sequencing (NGS) data of individuals from January 2016 to June 2020 for 17q12 duplication. To identify the function of the key gene of *HNF1B* in the 17q12 duplication region, human *HNF1B* mRNA was microinjected into LiPan zebrafish transgenic embryos. A total of 19 cases of 17q12 duplication were confirmed. AP was diagnosed during exploratory laparotomy in four patients (21.1%). The other common features of 17q12 duplication included intellectual disability (50%), gross motor delay (50%), and seizures/epilepsy (31.58%). The ratio of the abnormal pancreas in zebrafish was significantly higher in the *HNF1B* overexpression models. In conclusion, we first reported AP in patients with duplication of the 17q12 region, resulting in the phenotype of 17q12 duplication syndrome. Furthermore, our zebrafish studies verified the role of the *HNF1B* gene in pancreatic development.

## Introduction

Annular pancreas (AP) is a morphological anomaly that results in the pancreatic tissue completely or incompletely surrounding the duodenum (Kiernan et al., 1980). The incidence of AP is estimated to range from 0.015 to 0.05% (Maker et al., 2003; Rondelli et al., 2016). The common symptoms of AP are abdominal pain, vomiting, acute or chronic pancreatitis, and swollen belly (Zyromski et al., 2008). The degree of manifestation depends on the severity of the intestinal blockage.

AP has been reported to be associated with some congenital anomalies (Jimenez et al., 2004; Wang et al., 2018). Some chromosome disorders, such as Down’s syndrome, have been reported in 8–25% of patients with AP (Jimenez et al., 2004; Wang et al., 2018). Jacobsen syndrome has also been reported to be associated with AP (Fernández González et al., 2002). Chromosome 17q12 recurrent deletion syndrome (MIM: 614527), caused by the presence of a 1.4-Mb deletion at the approximate position of 36,458,167–37,854,616 (GRCh37/hg19), presents various clinical phenotypes including kidney anomalies, maturity-onset diabetes of the young type 5, and agenesis of the dorsal pancreas (Mitchel et al., 1993; Andersen and Schaffalitzky de Muckadell, 2019). Furthermore, chromosome 17q12 recurrent duplication syndrome (MIM: 614526), caused by a 1.4-Mb duplication at the approximate position of chr17:34,815,072–36,192,492 (GRCh37/hg19), presents various clinical phenotypes, including behavioral abnormalities, neurological symptoms, and brain abnormalities (Mefford et al., 1993; Mefford et al., 2007). However, AP has not been reported in either 17q12 deletion syndrome or 17q12 duplication syndrome.

There are 15 genes in the 17q12 recurrent duplication/deletion regions, namely, *ATF*, *ACACA*, *C17orf78*, *DDX52*, *DHRS11*, *DUSP14*, *GGNBP2*, *HNF1B*, *LHX1*, *MRM1*, *MYO19*, *PIGW*, *SYNRG*, *TADA2A*, and *ZNHIT3* (Mefford et al., 1993). Not all genes are haploinsufficient. Among these genes, variants in three genes (*ACACA*, *PIGW*, and *ZNHIT3*) can cause autosomal recessive inheritance diseases, whereas variants in *HNF1B* cause two autosomal dominant inheritance diseases including non-insulin-dependent diabetes mellitus (MIM: 125853) and renal cysts and diabetes syndrome (MIM: 137920). The *HNF1B* gene encodes a member of the homeodomain-containing superfamily of transcription factors and is regarded as an important transcription factor that controls the development of the pancreas (Coffinier et al., 1999).

The 17q12 recurrent deletion/duplication can be detected by array-based comparative genomic hybridization (aCGH), exome sequencing (ES) with copy number variant (CNV) calling, genome sequencing, or targeted deletion analysis. Our recent studies (Dong et al., 2020; Wang et al., 2020) have reported that next-generation sequencing (NGS) has good performance in detecting CNVs. In this study, we describe 19 patients with duplication of 17q12, 4 of whom present with AP as identified by NGS. Furthermore, functional studies were conducted in zebrafish. This study aimed to describe additional clinical characteristics and provide experimental data to understand 17q12 duplication.

## Materials and Methods

### NGS Data Collection and CNV Calling

This study retrospectively collected the NGS data of individuals referred to the Center for Molecular Medicine of the Children’s Hospital of Fudan University (CHFU) for genetic testing from January 1, 2016 to June 1, 2020. This study was approved by the CHFU Ethics Committee (2020-440).

Clinical exome sequencing (CES) data of 2,720 genes and ES data were included. Sequence data were aligned to the reference human genome (GRCh37/hg19). The detailed procedure was described in our previous study (Yang et al., 2019). This study developed an in-house CNV detection pipeline based on CANOES and HMZDelFinder and combined it with PhenoPro to prioritize phenotype-related genetic analysis (Li et al., 2019). The clinical significance of the CNVs was determined based on the following literature and genetic databases: UCSC Genome Browser^1^, DECIPHER^2^, and ClinGen^3^. Agilent SurePrint G3 aCGH and SNP 4 × 180 K microarray (Agilent Technologies, United States) were used to confirm the CNVs detected by NGS following the manufacturer’s instructions. Data were processed using the DNA analytics software (Agilent Cytogenomics 4.0).

### Clinical Information Collection and Patient Follow-Up

The inclusion criteria of individuals were as follows: (1) 17q12 duplication detected using NGS data analysis and (2) 17q12 duplication confirmed using aCGH. Clinical information was collected from medical records and *via* phone-call follow-up.

### *In vitro* Study of the *HNF1B* Gene in Zebrafish

#### *In vitro* Synthesis of Wild-Type Human *HNF1B* and GFP mRNAs

The coding region of human *HNF1B* (NM_000458), which was synthesized by a biotechnology company (TsingKe, Beijing, China), and green fluorescent protein (GFP) was inserted into recombinant plasmids (pCS2+). Furthermore, 3 μg of each plasmid was digested with Not I. The insert containing the *HNF1B* or GFP cDNA was gel-purified and transcribed with SP6 RNA polymerase using the mMESSAGE mMACHINE^TM^ SP6 *in vitro* transcription kit (AM1340; Invitrogen) according to the manufacturer’s instructions. The mRNA was diluted with diethylpyrocarbonate-treated water at a final concentration of 100 ng/μl and stored at -80°C until use.

#### Microinjection of Zebrafish Eggs

Zebrafish eggs were obtained by random mating of LiPan zebrafish with wild-type zebrafish (TU strain). *In vitro* synthesized *HNF1B* and GFP mRNAs were injected into embryos at the 1–2-cell stage together with the dye tracer phenol red solution (P0290; Sigma). After microinjection, embryos were maintained in egg water with ∼0.0005% methylene blue in a standard laboratory environment (28.5°C) and a 14-h light/10-h dark cycle according to a standard protocol (Kalueff et al., 2014) until analysis. Egg water was refreshed every day, and embryos with obvious deformities were discarded.

#### Microscopy and Image Analysis

At 7 days post-fertilization, larvae with exocrine pancreas-specific GFP expression were selected for analysis. The larvae were anesthetized with 0.08% tricaine (E10521; Sigma) and immobilized in 3% methylcellulose (M0521; Sigma). Observations of live embryos were performed using a Nikon stereoscope (SMZ800N), and the number of zebrafish with abnormal pancreas was recorded. Photographs were obtained using a Leica confocal microscope (TCS-SP8), and the length of the pancreas was measured.

### Statistical Analysis

Data were presented as the mean ± standard error of the mean. Statistical analyses were performed, and graphs were plotted using the GraphPad Prism software (version 8.0). Student’s *t*-test (two-tailed) was used to analyze the changes between different larval groups. The minimal criterion of significance was set at P < 0.05.

## Results

### 17q12 Duplication Identified From NGS Data

**FIGURE 1:**
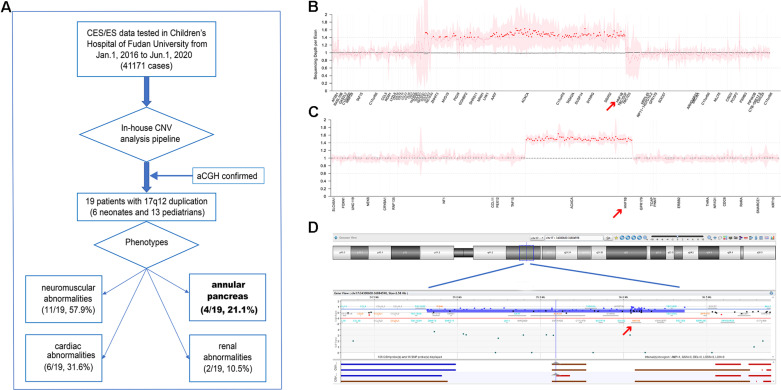
**(A)** Flow diagram of this study. **(B,C)** Normalized sequencing depth of exons in samples with 17q12 duplication, for exome sequencing (ES) **(B)** and clinical exome sequencing (CES) **(C)** samples, separately. Sequencing reads depth on each exon were normalized and summarized. The mean values were marked as dots (pink dots for normal regions and red dots for duplication regions), and 95% confidential interval was shown as pink shades. X-axis was re-scaled by exon numbers other than gene length. **(D)** 17q12 duplication verified in patient 5 by aCGH.

As shown in Figure 1A, a total of 41,171 individuals with suspected genetic diseases underwent genetic testing (CES or ES) in our laboratory from January 1, 2016 to June 1, 2020. All CES/ES data from these patients were analyzed for CNV calling. A total of 19 cases with 17q12 duplications were identified. Eight of these were tested using ES. Four cases (patients 7, 8, 11, and 15) had a 1,262.765-kb duplication (Figure 1B); however, in the remaining four cases, ES called four different sizes of duplication in the region of 17q12 (1,581.241 kb in patient 10, 1,532.705 kb in patient 6, 1,308.066 kb in patient 18, and 40.314 kb in patient 13). In the cases tested by CES, a 663.315-kb duplication (Figure 1C) was detected in all 11 cases (patients 1, 2, 3, 4, 5, 9, 12, 14, 16, 17, and 19). To confirm the size of the CNVs called by CES, aCGH was performed, and the length of the CNV in patient 5 was verified to be 1,516.456 kb (Figure 1D). All duplication regions included the *HNF1B* gene (Supplementary Figure 1). The detailed position of the 17q12 duplication in each patient is shown in Supplementary Table 1. No other pathogenic or likely pathogenic variants/CNVs were found in these 19 patients.

Six of the patients (31.58%) were neonates (<28 days), and 13 (68.42%) were pediatric patients (median age, 24 months). Among the 19 patients, AP was identified in 4 neonates (21.1%) who underwent exploratory laparotomy. The detailed clinical symptoms of the 19 patients are shown in Supplementary Table 2.

### Clinical Characteristics of Four Patients With AP

**FIGURE 2:**
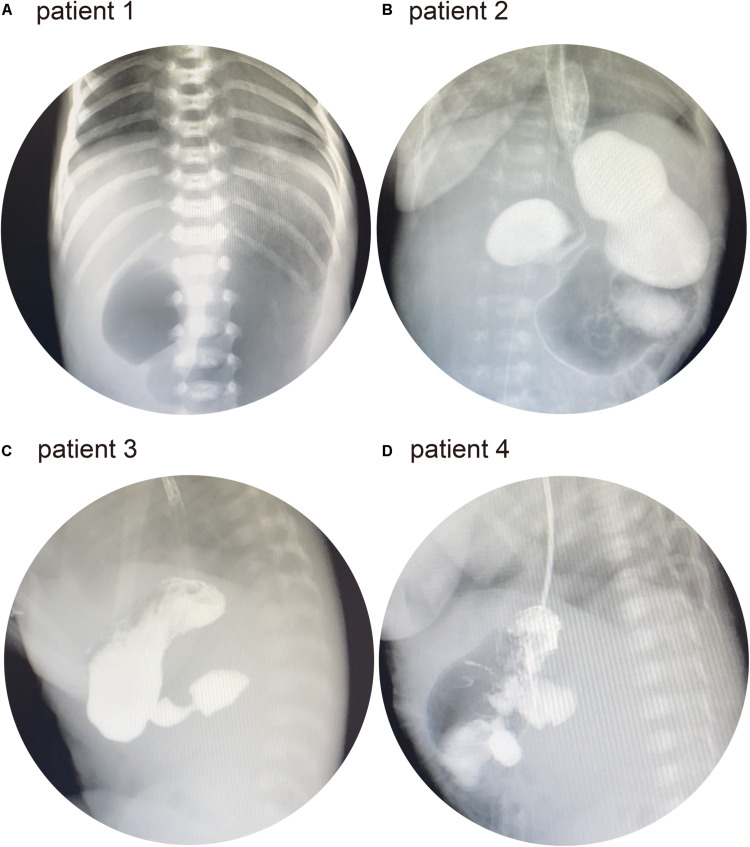
**(A)** Patient 1: abdominal X-ray showed the “double bubble” sign. **(B)** Patient 2: upper gastrointestinal series (UGI) showed distended stomach and duodenal bulb. **(C)** Patient 3: UGI showed a distended duodenal bulb. **(D)** Patient 4: UGI showed a distended duodenal bulb.

Patient 1 was the first child of a non-consanguineous couple. The breathing of the patient stopped twice at the age of 3 days. He was then presented to our hospital for further treatment. Physical examination revealed a yellowish skin color. Serum biochemistry tests showed a high level of direct bilirubin (34.5 μmol/L; normal range: 0–6.8 μmol/L) and total bilirubin (231.4 μmol/L; normal range: 0–17.1 μmol/L). Plain abdominal radiography revealed a duodenal ileus (Figure 2A). Electroencephalography showed that the patient experienced neonatal seizures. Moreover, he was diagnosed with renal abnormalities. Facial features were normal. In addition, there were no ophthalmologic, endocrine, and cardiac abnormalities in this patient. Subsequently, the patient was diagnosed with AP during exploratory laparotomy. The patient did not present with intellectual disability, gross motor delay, and behavioral abnormalities during follow-up.

Patient 2 was a 28-day-old male infant. The patient was presented to a local hospital because of vomiting at the age of 3 days. He had been diagnosed with duodenal ileus by upper gastrointestinal contrast study and underwent surgery. However, the patient did not recover and was transferred to our hospital for further treatment. Physical examination revealed normal facial features. The upper gastrointestinal series (UGI) showed duodenal ileus (Figure 2B). No other abnormalities were observed in patient 2. At the age of 32 days, he underwent exploratory laparotomy in our hospital and was diagnosed with AP.

Patient 3 was a 16-day-old male infant who was born *via* cesarean section. The patient was presented to our hospital owing to vomiting since birth. He had normal facial, ophthalmologic, and endocrine features, and the UGI showed a duodenal ileus (Figure 2C). Echocardiography revealed a patent arterial duct and a patent foramen ovale. At the age of 20 days, he underwent exploratory laparotomy and was diagnosed with AP.

Patient 4 was a 3-h-old male infant who was born *via* cesarean section. Prenatal color ultrasound indicated that the patient had duodenal ileus. Thus, the patient was transferred to our hospital for further treatment after birth. He had normal facial features. Ophthalmologic and endocrine tests were normal. Moreover, there was no abnormal cardiac malformation. His UGI revealed duodenal ileus (Figure 2D). The patient had been diagnosed with AP during surgery at the age of 5 days.

### Clinical Characteristics of Patients With 17q12 Duplication Detected in This Study and in the Literature

Among the 19 patients, different neurodevelopmental abnormalities were observed. Among the 10 patients available for intelligence quotient and language assessment, only 5 patients (5/10, 50%) were presented with intellectual disability, and 1 patient (1/10, 10%) had speech delay. Moreover, information on gross development could be obtained from 12 patients, and half of them (6/12, 50%) showed gross motor delay. Other neurodevelopmental abnormalities included seizures/epilepsy (6/19, 31.58%), hypotonia (2/19, 10.53%), and behavioral abnormalities (2/19, 10.53%). Some patients had congenital abnormalities. Six patients (6/19, 31.58%) had cardiac abnormalities, four patients (4/19, 21.1%) had AP, two patients had renal abnormalities (2/19, 10.53%), and one patient (1/19, 5.26%) had dysmorphic facial features. Moreover, three patients (3/19, 15.79%) had ophthalmologic abnormalities, and none of them had endocrine abnormalities. The detailed clinical characteristics of the patients are shown in Supplementary Table 1.

In addition, the clinical symptoms of patients with 17q12 duplication were reviewed and summarized. A total of 108 patients were included in the analysis. Among them, 75% (60/80) were children (>28 days–18 years), and 23.75% were adults (19/80) (>18 years). As shown in Table 1, intellectual disability (73.9%, 51/69) and speech delay (75.5%, 37/49) were the most common neurodevelopmental abnormalities. Other common findings included behavioral abnormalities (63.63%, 42/66) and hypotonia (57.9%, 11/19). The most common congenital abnormalities included dysmorphic facial features (59.6%, 34/57) and skeletal abnormalities (58.82%, 10/17). To the best of our knowledge, AP has not been reported in patients with 17q12 duplication.

**TABLE 1:** Summarized characteristics of patients with 17q12 duplication in the present study and published studies.

	19 Patients in this study (%)	108 Published patients (%)
**Sex**		
Male	12/19 (63.16)	52/94 (55.32)
Female	7/19 (36.84)	42/94 (44.68)
Age		
Neonate (0–28 days)	6/19 (31.58)	1/80 (1.25)
Children (>28 days–18 years)	13/19 (68.42)	60/80 (75)
Adult (>18 years)	0 (0)	19/80 (23.75)
**Clinical characteristics**		
**Neurodevelopmental abnormalities**		
Intellectual disability	5/10 (50.00)	51/69 (73.91)
Speech delay	1/10 (10.00)	37/49 (75.51)
Gross motor delay	6/12 (50.00)	30/53 (56.60)
Behavioral abnormalities	2/19 (10.53)	42/66 (63.63)
Seizures/epilepsy	6/19 (31.58)	30/59 (50.85)
Hypotonia	2/19 (10.53)	11/19 (57.89)
**Structure abnormalities**		
Dysmorphic facial features	1/19 (5.26)	34/57 (59.65)
Skeletal abnormalities	0/19 (0)	10/17 (58.82)
Cardiac abnormalities	6/19 (31.58)	10/26 (38.46)
Renal abnormalities	2/19 (10.53)	17/44 (38.64)
Annular pancreas	4/19 (21.1)	0 (0)
Endocrine abnormalities	0/19 (0)	12/40 (30.00)
Ophthalmologic abnormalities	3/19 (15.79)	15/46 (32.61)

### Overexpression of *HNF1B* mRNA in Zebrafish Embryos

Human *HNF1B* mRNA was microinjected into LiPan transgenic embryos with GFP fluorescence in the exocrine pancreas (Korzh et al., 2008) at the 1-cell stage (50 pg/embryo). In addition, GFP mRNA was synthesized and injected into embryos to confirm that the *in vitro* synthesis system worked well and to exclude the possibility that the phenomenon observed in *HNF1B* overexpression zebrafish results from random microinjection. As expected, GFP expression was observed in zebrafish at 24 h post-fertilization, and pancreatic morphology was similar to that in groups without injection (data not shown). Thus, embryos without injection and embryos injected with GFP mRNA were used as controls for the experiment.

**FIGURE 3:**
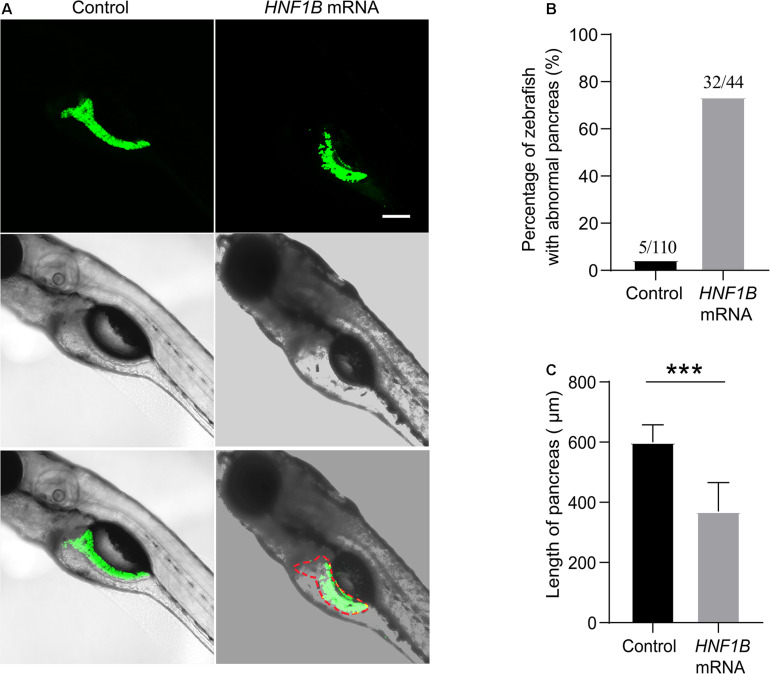
**(A)** Different features of zebrafish in the *HNF1B* overexpression group and the control group. **(B)** The ratio of zebrafish with abnormal pancreas in the *HNF1B* overexpression group and the control group. **(C)** The length of the pancreas in the *HNF1B* overexpression group and the control group.

Compared with the control, the morphology of the exocrine pancreas was obviously affected in the *HNF1B* overexpression groups. In the control group, the exocrine pancreas in zebrafish had a large anterior head region and a posterior tail elongated to the end of the yolk sac, whereas the exocrine pancreas in the *HNF1B* overexpression groups exhibited various abnormal morphologies, such as short pancreas and irregular or blurred shape of the pancreas (Figure 3A). In this study, we analyzed the ratio of zebrafish with an abnormal exocrine pancreas and the length of the pancreas. The ratio of zebrafish with abnormal pancreas was significantly higher (Figure 3B), and the length of the pancreas was significantly lower (Figure 3C) in the *HNF1B* overexpression groups than in the control group.

## Discussion

Individuals with AP may remain asymptomatic and may be diagnosed incidentally on imaging, surgery, or autopsy. However, a fraction of patients can present with intestinal obstruction in infancy or abdominal pain, peptic ulcer disease, and pancreatitis during adulthood (Sandrasegaran et al., 2009). In the present study, the symptoms of AP included vomiting and hyperbilirubinemia, which are common manifestations of AP (Wang et al., 2019). Imaging examination revealed duodenal ileus in four patients. Furthermore, all four patients were diagnosed with AP during surgery.

In 2006, Sharp et al. (2006) first reported chromosome 17q12 duplication in a patient with intellectual disability. The prevalence of 17q12 duplication syndrome is estimated to range from 0.037 to 0.25% (Stefansson et al., 2014; Mitchell et al., 2015). Early in 2001, Sun and Hopkins (2001) identified that the loss of *HNF1B* can lead to underdeveloped pancreas in zebrafish embryos. A subsequent study (Haumaitre et al., 2005) showed that a lack of *HNF1B* causes pancreatic agenesis in mice. Further functional research found that deletion of *HNF1B* can decrease proliferation and increase apoptosis in pancreatic multipotent progenitor cells, which leads to severe pancreatic hypoplasia and perinatal lethality (De Vas et al., 2015). In the *Xenopus laevis* model, overexpression of *Hnf1b* can lead to expansion of the pancreatic progenitor domain, but it has a limited influence on other genes in the adjoining region (Gere-Becker et al., 2018). This study used LiPan transgenic zebrafish embryos with GFP fluorescence in the exocrine pancreas to help visualize the development of the pancreas. Human *HNF1B* mRNA was microinjected into LiPan transgenic embryos to analyze the function of the *HNF1B* gene in pancreatic development. Compared with blank injection, *HNF1B* mRNA zebrafish showed various abnormal morphologies and a short length of the pancreas. Our data confirmed that overexpression of *HNF1B* plays a vital role in the development of the pancreas. Detailed mechanisms should be explored in the future.

To date, 108 patients with 17q12 duplication syndrome have been reported. The clinical characteristics of patients with 17q12 duplication are summarized in Table 1, and the details are reviewed in Supplementary Table 1. This study reported 19 patients with 17q12 duplications. The prevalence in our study was 0.046% (19/41,171), which is similar to that reported in the literature (Stefansson et al., 2014; Mitchell et al., 2015). The diagnostic age of patients in this study was less than that of the 108 patients reported in previous studies. All 19 patients were children, 6 of them were neonates, the median age was 24 months, and the average age was 50 months in the other 13 pediatric patients. Therefore, the evaluation of neurodevelopmental abnormalities related to intellectual disability, speech delay, and gross motor delay could only be performed in some patients. The behavioral abnormalities of some patients may occur later, and further examinations or follow-up is needed for the younger patients, particularly for the six neonates.

The diagnosed congenital abnormalities of dysmorphic facial features (59.65 vs. 5.26%), skeletal abnormalities (58.82 vs. 0%), and renal abnormalities (38.64 vs. 10.53%) of 19 patients were lower than those of 108 reported patients. AP was identified in 4 of 19 neonates with 17q12 duplication in this study. However, AP was not present in 108 reported patients. As AP may remain asymptomatic, we think that AP cannot be excluded in the reported 108 patients with 17q12 duplication. Approximately 40% of patients with AP diagnoses are diagnosed at surgery (Urayama et al., 1995), similar to our patients. Barr et al. (2020) reported an 11-year-old girl with DiGeorge syndrome who had been diagnosed with AP, even though the girl had a history of intermittent vomiting since birth. In this study, in 15 patients with 17q12 duplication, AP was not excluded. Thus, follow-up is necessary.

The duplication is recurrent and mediated by segmental duplications, and the reported size may be larger if adjacent segmental duplications are based on the design of the test method. However, although the capture regions of CES were different from those of ES, all the CNVs presented the key gene of 17q12 of *HNF1B*.

This study had two limitations. First, we only studied AP in patients with 17q12 duplication, but not with 17q12 deletion. The AP phenotype has not yet been identified. Second, the number of patients presented with the AP phenotype was only 4 of 19 with 17q12 duplication. In the other 15 patients, AP was not excluded, and follow-up was necessary for these patients to confirm if they had AP. Our future study will focus on whether the AP phenotype is present in patients with 17q12 deletion.

In conclusion, we first reported AP in patients with duplication of the 17q12 region that expanded the phenotype of 17q12 duplication syndrome. Further zebrafish studies have shown that the *HNF1B* gene plays an important role in the development of the pancreas.

## Data Availability Statement

The datasets used and/or analyzed during the current study are available from the corresponding authors on reasonable request.

## Ethics Statement

The studies involving human participants were reviewed and approved by Ethics Committee of Children’s Hospital of Fudan University. Written informed consent to participate in this study was provided by the participants’ legal guardian/next of kin. The animal study was reviewed and approved by Ethics Committee of Children’s Hospital of Fudan University. Written informed consent was obtained from the individual(s), and minor(s)’ legal guardian/next of kin, for the publication of any potentially identifiable images or data included in this article.

## Author Contributions

HW and WZ conceived and supervised the project. FX and XL designed and implemented the methods. YL, BW, RL, BL, KY, HC, GC, LW, QN, GL, PZ, XP, YC, and CS contributed to the data acquisition and analysis. HW, WZ, FX, and XL wrote the manuscript. All authors approved the manuscript.

## Conflict of Interest

The authors declare that the research was conducted in the absence of any commercial or financial relationships that could be construed as a potential conflict of interest.

## Footnotes

1 http://genome.ucsc.edu/

2 https://decipher.sanger.ac.uk/

3 https://clinicalgenome.org/

## Supplementary Material

Supplementary Figure 1 Locations of 17q12 duplication in our 19 patients.
