# Supplementary figures and images for: Overdosage of HNF1B Gene Associated With Annular Pancreas Detected in Neonate Patients With 17q12 Duplication
Source: Front Genet. 2021 May 7;12:615072. doi: 10.3389/fgene.2021.615072 (PMC8138176; doi:10.3389/fgene.2021.615072)

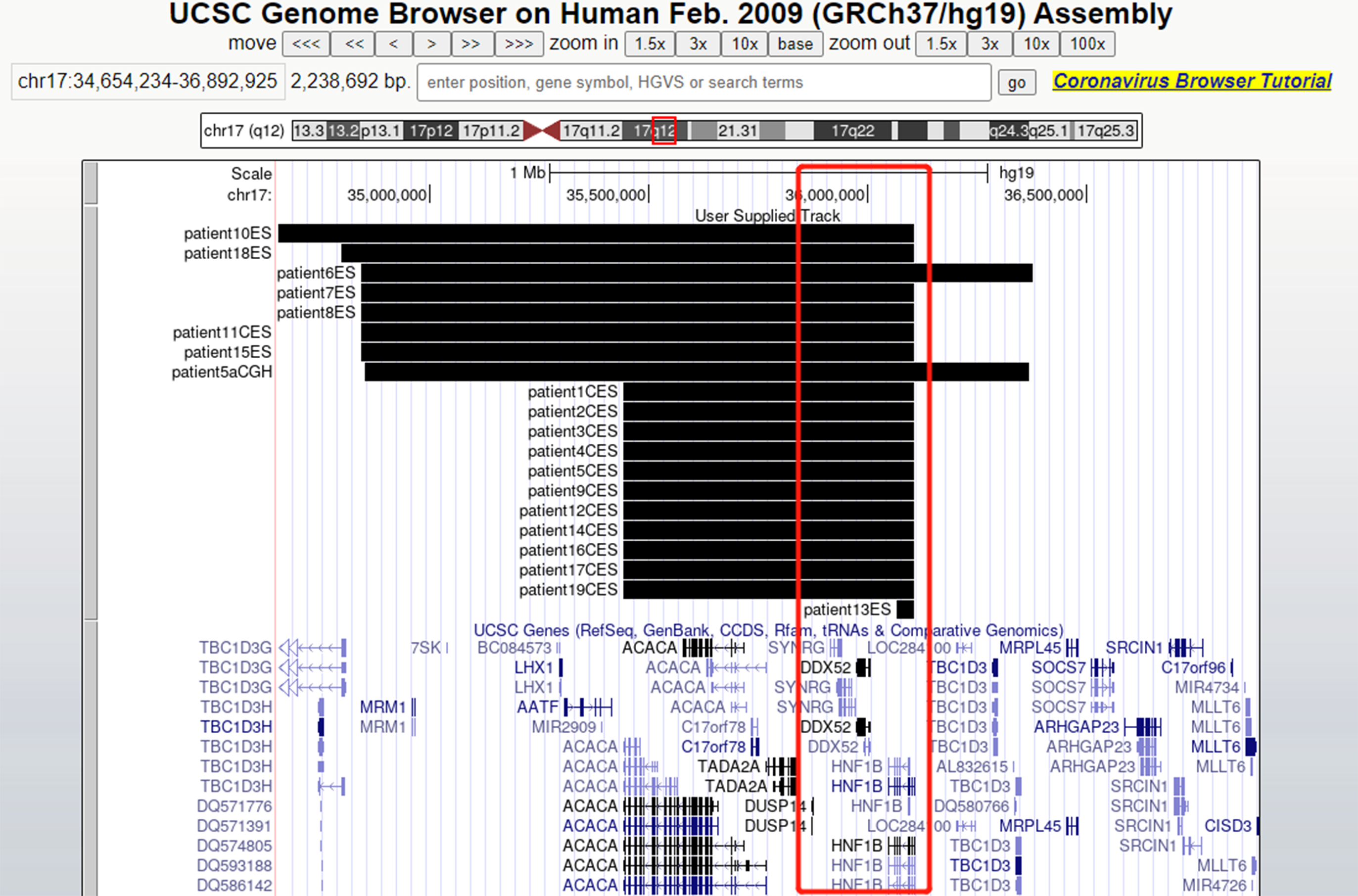

Supplement: Supplementary Figure 1 — Locations of 17q12 duplication in our 19 patients. [file Image_1.TIF]
