# Supplementary material for: Overdosage of HNF1B Gene Associated With Annular Pancreas Detected in Neonate Patients With 17q12 Duplication
Source: Front Genet. 2021 May 7;12:615072. doi: 10.3389/fgene.2021.615072 (PMC8138176; doi:10.3389/fgene.2021.615072)
Supplement: Supplementary file 2 [file Table_1.docx]

**Supplementary Table 1**: The CNV size and location in our 19 patients.

| Patient’s number | Methods | Chr | Regions | CNV size |
| --- | --- | --- | --- | --- |
| patient 1 | CES | 17 | 35441922-36105237 | 663.315 |
| patient 2 | CES | 17 | 35441922-36105237 | 663.315 |
| patient 3 | CES | 17 | 35441922-36105237 | 663.315 |
| patient 4 | CES | 17 | 35441922-36105237 | 663.315 |
| patient 5 | CES | 17 | 35441922-36105237 | 663.315 |
| patient 5 | aCGH | 17 | 34851066-36367522 | 1516.456 |
| patient 6 | ES | 17 | 34842472-36375177 | 1532.705 |
| patient 7 | ES | 17 | 34842472-36105237 | 1262.765 |
| patient 8 | ES | 17 | 34842472-36105237 | 1262.765 |
| patient 9 | CES | 17 | 35441922-36105237 | 663.315 |
| patient 10 | ES | 17 | 34523996-36105237 | 1581.241 |
| patient 11 | ES | 17 | 34842472-36105237 | 1262.765 |
| patient 12 | CES | 17 | 35441922-36105237 | 663.315 |
| patient 13 | ES | 17 | 36064923-36105237 | 40.314 |
| patient 14 | CES | 17 | 35441922-36105237 | 663.315 |
| patient 15 | ES | 17 | 34842472-36105237 | 1262.765 |
| patient 16 | CES | 17 | 35441922-36105237 | 663.315 |
| patient 17 | CES | 17 | 35441922-36105237 | 663.315 |
| patient 18 | ES | 17 | 34797171-36105237 | 1308.066 |
| patient 19 | CES | 17 | 35441922-36105237 | 663.315 |

Annote: CNV, copy number variation; CES, clinical exome sequencing; ES, exome sequencing; aCGH, array-based comparative genomic hybridization.
