# Supplementary material for: Overdosage of HNF1B Gene Associated With Annular Pancreas Detected in Neonate Patients With 17q12 Duplication
Source: Front Genet. 2021 May 7;12:615072. doi: 10.3389/fgene.2021.615072 (PMC8138176; doi:10.3389/fgene.2021.615072)
Supplement: Supplementary file 3 [file Table_2.DOCX]

Supplementary Table 2: Clinical characteristics of 108 published patients and 19 patients in the present study

| Patient's number | Age | Sex | ID | DFF | SD | GMD | BA | SA | Seizures/epilepsy | Hypotonia | Other^*^ | OA | EA | CA | RA | AP | PMID |
| --- | --- | --- | --- | --- | --- | --- | --- | --- | --- | --- | --- | --- | --- | --- | --- | --- | --- |
| 1 | - | - | Yes | - | - | - | - | - | - | - | - | - | - | - | - | - | 16906162 |
| 2 | - | - | Yes | - | - | - | - | - | - | - | - | - | - | - | - | - | 16906162 |
| 5812 | - | Male | Yes | - | - | - | - | - | Yes | - | Yes | - | None | - | - | - | 17924346 |
| 6498 | - | Female | Yes | - | - | - | - | - | Yes | - | Yes | - | None | - | - | - | 17924346 |
| 5858 | - | Female | - | - | - | - | - | - | Yes | - | - | - | - | - | - | - | 17924346 |
| 6880 | - | Male | - | - | - | - | - | - | Yes | - | - | - | - | - | - | - | 17924346 |
| IMR379 | - | Female | Yes | - | - | - | - | - | - | - | - | - | None | - | - | - | 17924346 |
| case 6 | 15 yrs | Male | Yes | Yes | - | - | - | - | - | - | - | Yes | - | Yes | - | - | 18657637 |
| case 6's sister | - | Female | - | - | - | - | Yes | - | - | - | None | None | None | - | - | - | 18657637 |
| Patient 5 | 4 yrs and 6 m | Male | - | None | - | None | - | - | None | None | None | - | None | - | None | - | 19844256 |
| Patient 6 | 4 yrs and 6 m | Male | - | None | - | Yes | - | - | None | None | None | - | - | - | - | - | 19844256 |
| Patient 7 | 45 yrs | Female | - | None | - | Yes | - | - | None | None | - | - | - | - | - | - | 19844256 |
| Patient 8 | 4 yrs and 4 m | Female | - | None | - | Yes | - | - | None | None | - | - | - | - | - | - | 19844256 |
| Patient 9 | 3 yrs | Male | - | Yes | - | Yes | - | - | None | None | - | - | - | - | - | - | 19844256 |
| patient | 29 yrs | Male | - | - | - | - | - | - | - | - | - | - | - | - | Yes | - | 21540130 |
| patient's child | child | - | - | None | - | - | - | - | - | - | None | - | - | None | Yes | - | 21540130 |
| patient | 5 yrs | Male | Yes | Yes | Yes | Yes | Yes | - | None | - | Yes | None | - | - | None | - | 22488896 |
| Family A: II-2 | - | Female | None | - | - | - | - | - | Yes | - | - | - | - | - | - | - | 24049133 |
| Family A: II-5 | - | Male | Yes | - | - | - | - | - | Yes | - | Yes | - | - | - | - | - | 24049133 |
| Family A: III-2 | - | Female | None | - | - | - | - | - | Yes | - | - | - | - | - | - | - | 24049133 |
| Family A: III-4 | - | Female | None | - | - | - | - | - | Yes | - | None | - | - | - | - | - | 24049133 |
| Family A: IV-2 | - | Male | Yes | - | - | - | - | - | Yes | - | None | - | - | - | - | - | 24049133 |
| Family B:I-2 | - | Male | None | - | - | - | - | - | Yes | - | - | - | - | - | - | - | 24049133 |
| Family B:II-2 | - | Male | None | - | - | - | - | - | Yes | - | - | - | - | - | - | - | 24049133 |
| Family B:III-3 | - | Female | None | - | - | - | - | - | Yes | - | - | - | - | - | - | - | 24049133 |
| 1 | 2 yrs | Male | - | Yes | - | Yes | None | Yes | - | Yes | - | - | - | - | - | - | 24239950 |
| NA | fetus | - | - | - | - | - | - | - | - | - | Yes | - | - | None | None | - | 25510704 |
| Patient 1 | 14 yrs | Female | - | - | - | None | Yes | - | - | - | Yes | Yes | Yes | - | Yes | - | 25691423 |
| Patient 2 | 13 yrs | Male | Yes | - | Yes | Yes | - | - | - | - | None | - | Yes | - | - | - | 25691423 |
| Patient | 5 yrs | Male | Yes | Yes | Yes | Yes | - | None | Yes | Yes | Yes | - | - | - | None | - | 23307502 |
| 11002.p1 | - | - | - | - | - | - | - | - | - | - | Yes | - | - | - | - | - | 23375656 |
| Patient V1 | 5 yrs | Male | Yes | - | Yes | Yes | Yes | - | - | - | - | - | - | None | - | - | 26123568 |
| Patient V2 | child | Female | Yes | - | Yes | - | - | - | - | - | - | - | - | None | - | - | 26123568 |
| Patient V3 | neo-te | Female | - | - | - | - | - | - | - | - | - | - | - | Yes | - | - | 26123568 |
| Case 1 | 7 yrs | Female | Yes | Yes | - | Yes | - | - | - | - | Yes | - | Yes | None | None | - | 26420380 |
| Case 2 | 26 m | Male | None | Yes | Yes | None | - | None | None | - | - | None | - | - | - | - | 26420380 |
| Case 3 | 16 m | Female | Yes | Yes | - | - | - | - | - | - | - | - | - | - | None | - | 26420380 |
| Case 4 | 6 yrs | Male | Yes | - | Yes | - | - | - | - | - | - | - | - | - | - | - | 26420380 |
| Case 5 | 9 yrs | Male | Yes | Yes | Yes | Yes | - | - | - | Yes | - | None | - | - | - | - | 26420380 |
| Case 6 | 25 yrs | Male | Yes | Yes | Yes | None | - | Yes | Yes | - | - | - | - | - | - | - | 26420380 |
| Case 7 | 24 yrs | Male | Yes | - | - | - | Yes | - | - | - | - | - | - | - | - | - | 26420380 |
| Case 8 | 14 yrs | Male | Yes | Yes | - | Yes | - | - | - | - | - | Yes | - | None | None | - | 26420380 |
| Case 9 | 15 yrs | Male | Yes | Yes | Yes | Yes | - | - | - | - | - | None | - | None | None | - | 26420380 |
| Case 10 | 8 yrs | Female | Yes | - | Yes | Yes | Yes | None | - | - | - | None | - | None | None | - | 26420380 |
| Case 11 | 17 m | Male | - | Yes | Yes | None | - | - | - | - | - | - | - | - | - | - | 26420380 |
| Case 12 | 2.5 m | Female | - | Yes | - | - | - | - | - | - | - | Yes | - | Yes | Yes | - | 26420380 |
| Case 13 | 18 yrs | Female | Yes | Yes | - | Yes | - | Yes | - | Yes | Yes | None | Yes | None | Yes | - | 26420380 |
| Case 13a | 18 yrs | Female | Yes | Yes | - | - | - | Yes | - | Yes | Yes | Yes | Yes | - | Yes | - | 26420380 |
| Case 14 | 3.5 yrs | Male | Yes | Yes | - | Yes | - | Yes | - | Yes | - | - | Yes | - | - | - | 26420380 |
| Case 15 | 4.5 yrs | Male | - | None | Yes | None | - | - | Yes | - | Yes | - | - | - | - | - | 26420380 |
| Case 16 | 13 yrs | Female | Yes | - | - | - | - | - | - | - | Yes | - | - | - | - | - | 26420380 |
| Case 17 | 11 yrs | Male | None | Yes | None | None | - | Yes | None | - | - | Yes | - | Yes | - | - | 26420380 |
| Case 18 | 9 yrs | Male | Yes | None | Yes | Yes | - | Yes | - | None | - | - | - | - | - | - | 26420380 |
| Case 18a | 16 yrs | Female | Yes | None | Yes | - | - | - | Yes | - | - | - | - | - | - | - | 26420380 |
| Case 19 | 3 yrs | Male | Yes | None | None | None | - | None | Yes | - | - | None | - | None | None | - | 26420380 |
| Case 20 | 3 yrs | Male | Yes | Yes | Yes | None | - | None | None | None | Yes | Yes | - | Yes | None | - | 26420380 |
| Case 21 | 14 yrs | Male | Yes | Yes | Yes | Yes | Yes | Yes | Yes | None | Yes | Yes | - | Yes | None | - | 26420380 |
| Case 22 | 15 yrs | Male | Yes | Yes | Yes | None | Yes | Yes | Yes | Yes | - | - | - | - | None | - | 26420380 |
| Case 23 | 7 yrs | Male | Yes | Yes | Yes | Yes | - | - | Yes | Yes | Yes | Yes | Yes | Yes | Yes | - | 26420380 |
| Case 24 | 4 yrs | Male | Yes | None | Yes | - | Yes | - | - | - | - | None | - | None | None | - | 26420380 |
| Case 25 | 11 yrs | Female | Yes | - | Yes | Yes | Yes | Yes | - | - | - | None | - | None | None | - | 26420380 |
| Case 26 | 8 m | Female | - | Yes | - | - | - | Yes | - | - | - | None | - | None | None | - | 26420380 |
| Case 27 | 5 yrs | Female | Yes | Yes | Yes | Yes | Yes | Yes | - | Yes | - | None | None | None | None | - | 26420380 |
| Case 28 | 16 m | Female | - | Yes | Yes | None | None | - | Yes | - | - | None | - | None | None | - | 26420380 |
| Case 29 | 9 yrs | Male | Yes | None | Yes | - | Yes | - | - | - | - | - | - | - | - | - | 26420380 |
| Case 30 | 9 yrs | Male | Yes | None | Yes | None | Yes | - | Yes | - | - | None | - | None | - | - | 26420380 |
| Patient | 19 yrs | Male | Yes | - | Yes | - | Yes | - | Yes | - | Yes | - | - | - | - | - | 26582588 |
| Patient | - | - | - | - | - | - | - | - | - | - | Yes | - | - | - | - | - | 20967226 |
| Patient | 4 yrs | Female | - | - | - | Yes | - | - | Yes | - | - | Yes | - | - | - | - | 24780443 |
| Patient's father | - | Male | - | - | - | - | - | - | - | - | Yes | - | - | - | - | - | 24780443 |
| Patient's sibling | - | Female | - | - | - | - | - | - | - | - | Yes | - | - | - | - | - | 24780443 |
| case II-2 | fetus | - | - | - | - | - | - | - | - | Yes | Yes | - | - | - | Yes | - | 32519823 |
| WG2716 | 19 m | Male | - | - | - | - | - | - | - | Yes | - | - | Yes | - | - | - | 31462756 |
| NA | - | - | - | - | - | - | - | - | - | - | - | - | - | - | Yes | - | 30578417 |
| NA | - | - | - | - | - | - | - | - | - | - | - | - | - | - | Yes | - | 30578417 |
| NA | - | - | - | - | - | - | - | - | - | - | - | - | - | - | Yes | - | 30578417 |
| NA | - | - | - | - | - | - | - | - | - | - | - | - | - | - | Yes | - | 30578417 |
| NA | - | - | - | - | - | - | - | - | - | - | - | - | - | - | Yes | - | 30578417 |
| NA | - | - | - | - | - | - | - | - | - | - | - | - | - | - | Yes | - | 30578417 |
| patient no. 0033 | 3 m | - | - | - | - | - | - | - | - | - | - | - | - | Yes | - | - | 27930557 |
| patient III-3 | 20 yrs | Male | Yes | Yes | Yes | Yes | - | Yes | - | - | Yes | Yes | - | - | - | - | 30134084 |
| patient II-2 | 46 yrs | Female | None | Yes | Yes | None | - | - | - | - | - | - | Yes | Yes | Yes | - | 30134084 |
| patient I-2 | 70 yrs | Female | None | Yes | - | - | Yes | - | Yes | - | Yes | - | Yes | - | None | - | 30134084 |
| patient II-4 | 43 yrs | Male | Yes | Yes | Yes | None | Yes | - | Yes | - | Yes | - | Yes | - | None | - | 30134084 |
| patient III-2 | 27 yrs | Female | Yes | Yes | - | - | Yes | - | Yes | - | Yes | - | - | - | None | - | 30134084 |
| NA | 17 yrs | Male | None | - | - | - | None | - | None | - | None | - | None | Yes | None | - | 28940454 |
| patient 1.1 | 9 m | Male | - | None | - | Yes | - | None | None | - | None | None | None | - | None | - | 27409573 |
| patient 1.2 | 22 yrs | Female | - | - | None | - | - | None | None | - | None | None | None | - | - | - | 27409573 |
| patient 2.1 | 6 yrs | Male | Yes | Yes | Yes | Yes | - | None | None | - | None | Yes | None | - | None | - | 27409573 |
| patient 3.1 | 12 yrs | Female | Yes | None | Yes | Yes | - | Yes | Yes | - | Yes | None | None | - | - | - | 27409573 |
| patient 4.1 | 4yrs | Female | - | Yes | None | Yes | - | Yes | Yes | - | Yes | None | None | - | None | - | 27409573 |
| patient 5.1 | 8 yrs | Female | Yes | None | Yes | None | - | None | None | - | None | Yes | None | - | None | - | 27409573 |
| patient 5.2 | 5 yrs | Female | None | None | None | None | - | None | None | - | None | None | None | - | - | - | 27409573 |
| patient 5.3 | 29 yrs | Female | Yes | None | None | None | - | None | None | - | None | None | None | - | - | - | 27409573 |
| patient 6.1 | 1 month | Male | - | None | - | - | - | None | None | - | None | None | None | - | - | - | 27409573 |
| patient 6.2 | 8 yrs | Male | None | None | Yes | None | - | None | None | - | autism | None | None | - | - | - | 27409573 |
| patient 6.3 | 41 yrs | Male | None | None | None | None | - | None | None | - | None | None | None | - | - | - | 27409573 |
| patient 7.2 | 28 yrs | Female | Yes | - | Yes | None | - | None | None | - | None | None | None | - | - | - | 27409573 |
| patient 7.3 | 25 yrs | Female | Yes | - | None | None | - | None | None | - | None | None | None | - | - | - | 27409573 |
| patient 7.4 | 54 yrs | Male | None | - | None | - | - | None | None | - | None | None | None | - | - | - | 27409573 |
| patient 8.1 | 14 yrs | Male | Yes | - | Yes | Yes | - | None | None | - | None | None | None | - | - | - | 27409573 |
| patient 9.2 | 34 yrs | Male | None | - | - | - | - | None | None | - | None | None | None | - | - | - | 27409573 |
| patient 10.1 | 19 yrs | Female | Yes | None | Yes | Yes | - | None | None | - | Yes | None | None | - | None | - | 27409573 |
| patient 11.1 | 2 m | Female | - | - | - | - | - | None | None | - | None | Yes | None | - | Yes | - | 27409573 |
| patient 11.2 | 27 yrs | Female | None | None | None | None | - | None | None | - | None | None | None | - | - | - | 27409573 |
| patient 12.1 | 1 year | Female | - | Yes | None | None | - | None | Yes | - | Yes | None | None | - | Yes | - | 27409573 |
| patient 13.1 | 12 yrs | Male | Yes | Yes | Yes | Yes | - | Yes | None | - | Yes | Yes | Yes | - | - | - | 27409573 |
| patient 13.2 | 44 yrs | Male | None | None | None | - | - | Yes | None | - | Yes | - | None | - | - | - | 27409573 |
| patient 1 | 3 days | male | None | None | None | None | None | None | Yes | None | None | None | None | None | Yes | Yes | This study |
| patient 2 | 28 days | male | - | None | - | - | None | None | None | None | None | None | None | None | None | Yes | This study |
| patient 3 | 16 days | male | - | None | - | - | None | None | None | None | None | None | None | Yes | None | Yes | This study |
| patient 4 | 0 day | male | - | None | - | - | None | None | None | None | None | None | None | None | None | Yes | This study |
| patient 5 | 2 yrs | female | Yes | None | None | Yes | None | None | None | None | None | None | None | None | None | None | This study |
| patient 6 | 2 yrs and 7 m | male | None | Yes | None | Yes | None | None | None | Yes | None | Yes | None | None | None | None | This study |
| patient 7 | 1 year and 4 m | male | Yes | None | None | Yes | None | None | Yes | None | Yes | Yes | None | Yes | None | None | This study |
| patient 8 | 34 days | male | - | None | - | None | None | None | None | None | None | None | None | None | None | None | This study |
| patient 9 | 14 yrs and 6 m | female | Yes | None | None | Yes | Yes | None | None | Yes | None | None | None | None | None | None | This study |
| patient 10 | 7-year | female | Yes | None | None | None | None | None | Yes | None | None | None | None | None | None | None | This study |
| patient 11 | 1 month | male | - | None | - | - | None | None | None | None | None | None | None | Yes | None | None | This study |
| patient 12 | 2 m | male | - | None | - | - | None | None | None | None | None | None | None | None | None | None | This study |
| patient 13 | 8-year | female | None | None | None | None | None | None | Yes | None | None | None | None | None | Yes | None | This study |
| patient 14 | 1 year and 9 m | male | Yes | None | Yes | Yes | None | None | None | None | None | None | None | Yes | None | None | This study |
| patient 15 | 5 yrs and 7 m | female | None | None | None | None | None | None | None | None | None | None | None | None | None | None | This study |
| patient 16 | 8 m | female | - | None | - | None | None | None | Yes | None | None | Yes | None | None | None | None | This study |
| patient 17 | 10 days | male | - | None | - | - | None | None | None | None | None | None | None | Yes | None | None | This study |
| patient 18 | 10 yrs and 5 m | male | None | None | None | Yes | None | None | Yes | None | None | None | None | None | None | None | This study |
| patient 19 | 21 days | female | - | None | - | - | None | None | None | None | None | None | None | Yes | None | None | This study |

**^*^**neurologic abnormalities; -: not available; NA: not available; none, no this feature; Yes, have this feature; yrs, years; m, months; ID, intellectual disability; DFF, dysmorphic facial features; SD, speech delay;

GMD, gross motor delay; BA, behavioral abnormalities; SA, skeletal abnormalities; OA, ophthalmologic abnormalities; EA, endocrine abnormalities; CA, cardiac abnormalities; RA, renal abnormalities;

AP, annular pancreas.
